# Ginsenoside Rh1 Alleviates Allergic Rhinitis by Mediating Mitochondrial Autophagy via Activation of the AMPK/ULK1/FUNDC1 Pathway

**DOI:** 10.1002/fsn3.70464

**Published:** 2025-06-17

**Authors:** Jiangang Wang, Yalin Zhang, Jingmei Chai, Jianing Yang, Longzhu Dai, Yi Yang, Yulian Zhang, Yongde Jin, Chongyang Wang, Guanghai Yan

**Affiliations:** ^1^ Jilin Key Laboratory for Immune and Targeting Research on Common Allergic Diseases Yanbian University Yanji P.R. China; ^2^ Department of Anatomy, Histology and Embryology Yanbian University Medical College Yanji P.R. China; ^3^ Department of Otolaryngology Head and Neck Surgery Affiliated Hospital of Yanbian University Yanji P.R. China; ^4^ Department of Traditional Chinese Medicine Yanbian University Medicine College Yanji P.R. China; ^5^ Key Laboratory of Natural Medicines of the Changbai Mountain, Ministry of Education Yanbian University Yanji P.R. China

**Keywords:** allergic rhinitis, AMPK/ULK1/FUNDC1 pathway, apoptosis, Ginsenoside Rh1, mitochondrial autophagy

## Abstract

Ginsenoside Rh1, a bioactive compound derived from ginseng, exhibits notable anti‐inflammatory and antioxidant effects and has shown promising therapeutic potential in the treatment of allergic diseases. However, its exact role in allergic rhinitis (AR) and the underlying molecular mechanisms remain inadequately understood. This study investigates whether Rh1 alleviates AR through AMPK/ULK1/FUNDC1‐mediated mitochondrial autophagy. In this study, human nasal epithelial cells (HNEpCs) were stimulated with house dust mite (HDM) and treated with mitochondrial autophagy inhibitors or siRNA transfection techniques to assess the effects of Rh1. Network pharmacology and molecular docking (MD) were used to explore the interactions between Rh1 and AMPK, ULK1, and FUNDC1. To explore the effects of Rh1, enzyme‐linked immunosorbent assay (ELISA) and flow cytometry (FC) were employed to measure IgE levels and various inflammatory mediators. Western blot (WB) analysis was conducted to assess protein expression related to mitochondrial autophagy, inflammation, and apoptosis in nasal tissues and HNEpCs. Immunofluorescence (IF) and transmission electron microscopy (TEM) provided further verification. The experimental data reveal that Rh1 effectively alleviates HDM‐induced nasal mucosal epithelial thickening and eosinophil infiltration by modulating mitochondrial autophagy via the AMPK/ULK1/FUNDC1 signaling pathway. Additionally, Rh1 inhibits IL‐4 secretion in nasal airway lavage fluid (NALF) and helps restore the Th1/Th2 immune balance. It also reduces mtROS production, inhibits NLRP3 inflammasome activation, and prevents apoptosis, thereby mitigating tissue damage associated with AR. Knockdown of AMPK or treatment with 3‐Methyladenine (3‐MA) further confirmed Rh1's inducing effect on mitophagy. In summary, Rh1 modulates mitophagy through the AMPK/ULK1/FUNDC1 pathway, reducing inflammatory responses and inhibiting apoptosis, thereby offering significant protection against AR.

## Graphical abstract

Ginsenoside Rh1 is involved in the inflammatory process of allergic rhinitis through the AMPK/ULK1/FUNDC1 signaling axis. Ginsenoside Rh1 can alleviate the pulmonary inflammation and apoptosis in asthmatic mice induced by house dust mite (HDM). Ginsenoside Rh1 regulates mitophagy and inhibits the NLRP3 inflammasome.

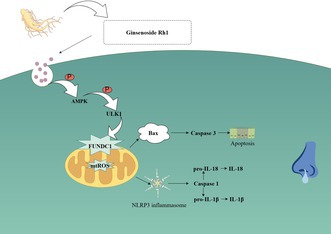

## 1 Introduction

AR is an immune‐mediated disorder, typically manifested by recurrent symptoms such as nasal congestion and nasal itching (Okubo et al. 2020). Current treatment methods, including antihistamines and topical corticosteroids, effectively alleviate symptoms but do not definitively cure the disease (Hoyte and Nelson 2018).

Studies have identified AR as an IgE‐mediated Type I hypersensitivity reaction, with Th2 immune responses mediating inflammation (Mandhane et al. 2011). Abnormal immune responses are closely related to immune cell dysfunction, mitochondrial function imbalance, and increased oxidative stress (OS) (Wu et al. 2023). Consequently, restoring mitochondrial homeostasis to regulate immune cell metabolism has emerged as a significant research focus for AR treatment (Li et al. 2023).

Ginsenosides, natural components of traditional Chinese medicine extracted from ginseng, include Ginsenoside Rh1, known for its neuroprotective and potential anti‐tumor effects, and utility in treating chronic inflammatory diseases (Tam et al. 2018). Previous studies have indicated that Ginsenoside Rh1 ameliorates type 2 diabetic nephropathy by modulating the AMPK/PI3K/AKT signaling pathway, thereby reducing inflammation and cell apoptosis (Su et al. 2021). Furthermore, Rh1 inhibits immune cell infiltration and prevents allergic asthma by blocking the activation of the MAPK, Akt, and NF‐κB pathways (Jin et al. 2023). Rh1 has also been shown to suppress migration and invasion in MDA‐MB‐231 cells by modulating STAT3/NF‐κB signaling through mitochondrial ROS (Jin et al. 2021).

AMP‐activated protein kinase (AMPK) acts as a cellular energy sensor, regulating a variety of physiological processes. It directly activates ULK1, which plays a pivotal role in cellular responses to nutrient deprivation and OS (Kim et al. 2011). AMPK mediates the phosphorylation of multiple ULK1 sites (Mihaylova and Shaw 2011), facilitating ULK1 translocation to mitochondria (Kim et al. 2011; Egan et al. 2011) and promoting the phosphorylation of FUNDC1, which regulates mitophagy (Wu et al. 2014). FUNDC1 is crucial for responding to energy crises, particularly through the AMPKα1/ULK1/FUNDC1 pathway, which promotes mitophagy in energy‐demanding tissues such as the heart (Cai et al. 2022) and nervous system (Zhang et al. 2023). Studies have shown that the gut microbiota's metabolism of FUNDC1‐mediated mitophagy improves inflammatory damage in kidney diseases (Li et al. 2024).

Research suggests that mitochondrial damage triggers the accumulation of PINK1 on the outer mitochondrial membrane, which recruits Parkin from the cytoplasm to depolarized mitochondria. This process initiates mitophagy through mitochondrial protein ubiquitination, thus providing cellular protection (Nguyen et al. 2016). Mitochondrial dysfunction has been closely linked to several allergic diseases, including allergic dermatitis and asthma (Albano et al. 2022). Mitophagy suppresses inflammation triggered by mitochondria, potentially playing a crucial role in regulating mitochondrial dysfunction and energy metabolism disorders (Melser et al. 2015). However, research on mitophagy in AR remains limited. Therefore, this study explores whether Ginsenoside Rh1 activates mitophagy via the AMPK/ULK1/FUNDC1 pathway to alleviate AR.

## 2 Materials and Methods

### 2.1 Mice

We obtained female C57 mice from Yanbian University's Experimental Animal Management Center. The mice were 6–8 weeks old and weighed 20–22 g. The mice were kept in a controlled environment with a 12‐h light/dark cycle, relative humidity of 50%–60%, and an ambient temperature of 22°C ± 2°C. In accordance with the “Regulations for the Administration of Experimental Animals” and with the consent of the Medical College of Yanbian University Ethics Committee (consent No.: YD20240927002), all experimental procedures were carried out.

### 2.2 Animal Handling and Grouping

Wild‐type mice were randomly assigned to five groups: Control, HDM‐induced AR model, HDM + Rh1 (25 mg/kg) (Tam et al. 2018), HDM + Rh1 (50 mg/kg) (Tam et al. 2018), and HDM + Dex (5 mg/kg) (Son et al. 2022), with 20 mice in each group. During the 28‐day experimental period, intranasal administration of 20 μL of HDM (1 mg/mL, Greer Laboratories, Lenoir, NC, USA) or an equal volume of phosphate‐buffered saline (PBS) occurred three times a week. The corresponding therapeutic drugs were administered 1 h prior to each exposure in the last week (Nakanishi et al. 2013). Ginsenoside Rh1 was employed (molecular formula: C36H62O9, Lu et al. 2017, molecular weight: 638.87, purity 98%, B21061, Shanghai YuanYe Biotech Co. Ltd., China). After the final exposure, the mice were observed for 24 h and then euthanized.

### 2.3 Assessment of Nasal Allergic Symptoms and Sample Collection in Mice

After the last HDM challenge, the number of sneezes and nose rubs within 15 min was recorded to evaluate nasal allergy reactions. Blood was drawn from the mice's orbits 24 h after they were euthanized. After centrifugation was used to extract serum, the nasal cavity was lavaged with PBS, and the fluid from the lavage was collected (NALF).

### 2.4 ELISA

The concentrations of cytokines (IL‐4, IL‐5, IL‐13, and TNF‐α) in mouse serum and bronchoalveolar lavage fluid (BALF) were quantified using commercial ELISA kits (Mlbio, Shanghai, China) according to the manufacturer's protocol. Briefly, thawed samples were centrifuged and diluted as needed. Working solutions, assay diluents, and standard curves were prepared fresh for each assay. Standards and samples were loaded in duplicate onto the pre‐coated 96‐well plate alongside blank controls. After a 2‐h incubation at 37°C, plates were washed three times with prepared wash buffer. Subsequently, 100× biotinylated detection antibody was diluted to 1× working concentration and added to each well for another 2‐h incubation at 37°C, followed by three washes. Horseradish peroxidase (HRP)‐conjugated streptavidin was then added and incubated for 1 h at 37°C. Following three final washes, tetramethylbenzidine (TMB) substrate was added for color development (20 min at 37°C in the dark), which was terminated by stop solution. Optical density at 450 nm was immediately measured using an Epoch microplate reader (BioTek), and cytokine concentrations were interpolated from standard curves generated with Excel software.

### 2.5 FC

Cell pellets were obtained by centrifuging NALF. Thermo Fisher Scientific's eBioscience Fixable Viability Dye eFluor 780 (65‐0865‐14) was used to stain the cells for viability monitoring. The following antibodies were used for staining: APC‐conjugated CD45.2 (#109814, Biolegend, USA), PerCP‐Cy5.5‐conjugated CD3e (45‐0031‐82, Thermo Fisher Scientific), and PE‐conjugated SiglecF (#155506, Biolegend). A Cytoflex S flow cytometer (Beckman Coulter Inc., Brea, CA, USA) was used to evaluate the samples after they had been stained in the dark at 4°C for 30 min.

### 2.6 Hematoxylin and Eosin Staining

The nasal tissues were preserved in 10% formaldehyde for 24 h at ambient temperature, then decalcified in EDTA for 8 to 12 days. After that, they were embedded in paraffin and sectioned at 4 μm. After dewaxing, the sections were rehydrated and dyed with hematoxylin and Eosin (H&E).

### 2.7 Terminal Deoxynucleotidyl Transferase dUTP Nick End Labeling Assay

To determine cell apoptosis, we used a Transferase dUTP Nick End Labeling (TUNEL) apoptosis detection kit (C1089, China). Before applying the TUNEL detection solution, we dewaxed and rehydrated the tissue sections. Then, we treated them with Deoxyribonuclease K (#ST532, Beyotime) at 37°C for 30 min. Finally, we analyzed the results using a Cytation 5 (Bio‐Tek Instruments, USA).

### 2.8 Cell Culture and Treatment

Geneo Biotech Co. Ltd. of Guangzhou, China, supplied the HNEpCs. For 1 h, the treatment groups were given either 25 or 50 μM of Rh1 (Wang et al. 2024) or 5 μM of Dex (von Mässenhausen et al. 2022).

During this period, HDM (10 μg/mL, Greer Laboratories) was added. After 24 h of transfection with Lipofectamine RNAiMAX Reagent (#13778030, Thermo Fisher Scientific, USA), cells expressing AMPK were silenced.

### 2.9 Detection of Mitochondrial Reactive OS

The DCFH‐DA fluorescent probe was used to evaluate the levels of intracellular ROS. For 30 min, cells were treated at 37°C with 5 μmol·L^−1^ of MitoSOX dye. The Cytation 5 imaging equipment was used for both observation and imaging.

### 2.10 Detection of Mitochondrial Membrane Potential

After a 20‐min incubation at 37°C, cells were dyed with JC‐1 (#C2006, Beyotime, Shanghai, China). Using the Cytation 5 imaging equipment for microscopy, the potential of the mitochondrial membrane was recorded.

### 2.11 Mitochondrial Isolation

Following ice homogenization of tissues and cells, mitochondria were separated using a Beyotime Mitochondrial Isolation Kit. Proteins from mitochondria were extracted by spinning samples at 12,000 × g for 10 min at 4°C.

### 2.12 Transmission Electron Microscopy Detection

Cells were fixed in 3% glutaraldehyde solution for 2 h and washed three times with PBS. Specimen preparation followed the standard operating procedures of the Pathology Department of Yanbian University. Images were captured at 6000× magnification using a transmission electron microscopy (TEM) (HT7700 Hitachi, Japan).

### 2.13 WB

Using RIPA lysis buffer (#P0013B, Beyotime, Shanghai), the cells and tissues in the nose that had been treated were lysed. An assay kit for BCA proteins (#P0010S, Beyotime, Shanghai) was used to measure the overall protein content. Membranes made of PVDF (Millipore, USA) were used after proteins were separated using 10% SDS‐PAGE. After being incubated with primary antibodies at 4°C for 2 h, followed by HRP‐conjugated secondary antibodies for 1 h, the membranes were blocked with 5% skim milk for 2 h. The process involved creating, imaging, and analyzing protein bands.

The primary antibodies used in this study are as follows: Bax (ab32503), Cyt‐c (ab133504), Bcl‐2 (ab194583), Caspase‐1 (ab207802), IL‐18 (ab243091), and p‐ULK1 (ab133766) were purchased from Abcam (USA); β‐Actin (3700), NLRP3 (DF7438), AMPK‐α (AF6423), p‐AMPK‐α (AF3423), PINK1 (DF7742), FUNDC1 (DF15481), p‐FUNDC1 (AF001), and p‐ULK1 (AF2301) were obtained from Affinity (USA); Bcl‐2‐related protein Bax (A19684), IL‐1β (A16288), ASC (A11433), and Parkin (A0968) were sourced from Abclonal (USA). Caspase‐3 (#9662), Cleaved‐Caspase‐1 (#89332S), Cleaved‐Caspase‐3 (#9644), and ULK1 (#8054) were purchased from Cell Signaling Technology (CST, USA). The optimal dilution ratio for each primary antibody was 1:1000.

The secondary antibodies used were HRP‐conjugated goat anti‐rabbit antibody (#5151, CST, USA) and HRP‐conjugated goat anti‐mouse antibody (#5257, CST, USA). The optimal dilution ratio for each secondary antibody was 1:5000.

### 2.14 Immunohistochemical Analysis

Paraffin sections were dewaxed and underwent antigen retrieval using heated citrate solution. Following washing, slices were treated with peroxidase and then with primary antibodies overnight at 4°C. After that, we added an enhancer solution and DAB, and then we incubated the samples with secondary antibodies. Following a mild hematoxylin counterstain, the sections were differentiated, dehydrated, and mounted.

### 2.15 IF

4% paraformaldehyde was used to fix HNEpCs, followed by 5% BSA to block them, and 0.2% Triton to permeabilize them. With the use of MitoTracker Red, PINK1, or Parkin, cells were stained overnight at 4°C. Alexa Fluor 488 goat anti‐rabbit and goat anti‐mouse IgG H&L were subsequently added, and the samples were incubated for 2 h at 37°C. An imaging system called Cytation 5 was used for fluorescence imaging.

Prior to incubation with primary antibodies, slices of nasal mucosa tissue were subjected to antigen retrieval. The samples were submerged in Alexa Fluor 488‐labeled goat anti‐rabbit and Alexa Fluor 568‐labeled goat anti‐mouse IgG H&L secondary antibodies for 1 h.

### 2.16 MD

Download the RH1B file from the RCSB database and preprocess it using pymol software, which includes removing water molecules and ions. Utilize Autodock software to hydrogenate the protein and calculate the charge. Compounds were downloaded as sdf files from Pubchem. Chem3Dpro software was used to calculate the minimum energy. Convert the sdf file to Rh1bqt format using Autodock and create 3D and 2D images with pymol. Generate 2D diagrams showcasing the docking sites using ligplot.

### 2.17 Network Pharmacology

Disease targets were sourced from the gene libraries of GeneCards, OMIM, and DISGENET; the compound structure and SMILES of the monomer were obtained from Pubchem. Data were imported into SwissTarget, PharmMapper, SEA, and SuperPred databases to identify targets.

### 2.18 Statistical Analysis

Data were analyzed using SPSS 19.0. Continuous variables were presented as mean ± standard deviation (SD), and categorical data were expressed as percentages (%). One‐way analysis of variance (ANOVA) was used for intergroup comparisons, while the *q* test was applied for intragroup comparisons. *p* < 0.05was considered statistically significant. *p < 0.05, **p < 0.01 compared to the control group; ^#^ *p* < 0.05,^##^ *p* < 0.01 compared to the HDM‐challenged group.

## 3 Results

### 3.1 Rh1 Alleviates Nasal Allergy‐Like Symptoms and Modulates Immune Response in Mice

**FIGURE 1:**
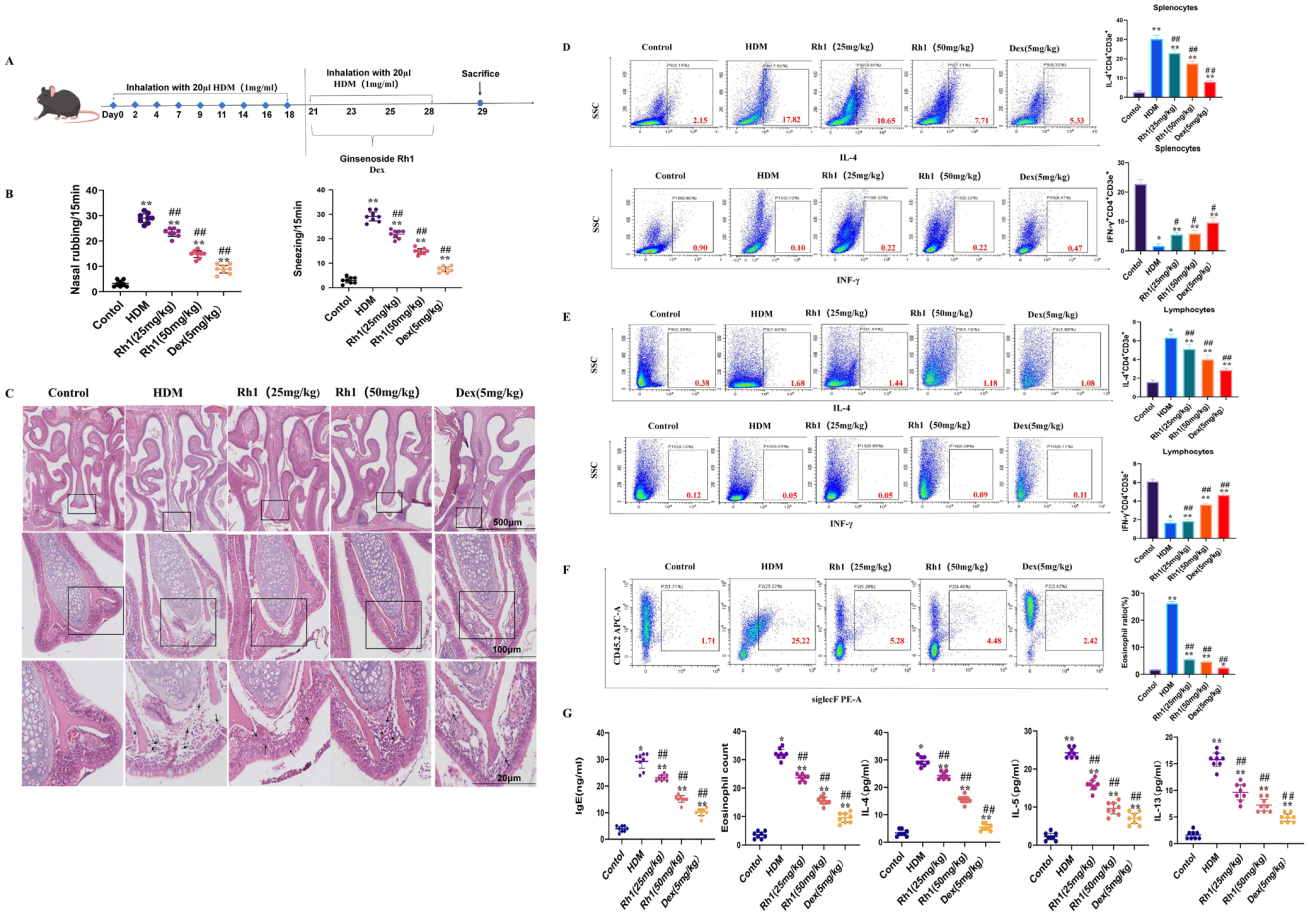
Inflammation and restoration of Th1/Th2 imbalance in AR mice. (A) Experimental protocol outlining treatment with HDM and PBS. (B) Frequency of sneezing and nasal rubbing observed in the mice. (C) HE staining of nasal mucosa tissues. (D–F) FC analysis depicting the ratio of CD4^+^ IL‐4^+^ cells to CD4^+^ IFN‐γ^+^ cells in the spleen, lymph nodes, and supernatant of NALF. (G) ELISA measurements of HDM‐specific IgE levels in serum, eosinophil count, and cytokine levels (IL‐4, IL‐5, and IL‐13) in the NALF supernatant. Data are presented as mean ± SD (*n* = 8). *p < 0.05, **p < 0.01 compared with control group. ^#^ *p* < 0.05,^##^ *p* < 0.01 compared with HDM‐challenged group.

A mouse model of AR was established through intranasal instillation of HDM (Figure 1A). Observations recorded within 15 min post‐final HDM challenge included sneezing and nose‐rubbing frequencies. These behaviors were significantly elevated in the model group (Figure 1B). Post‐Rh1 treatment, particularly at 50 mg/kg, a marked reduction in these frequencies was noted, demonstrating Rh1's potential to alleviate nasal allergy symptoms. H&E staining of nasal tissues showed thickening of the nasal mucosal epithelia and an increase in submucosal eosinophils in the model group, both of which were substantially reduced by Rh1 treatment (Figure 1C).

Allergen exposure prompted an increased secretion of inflammatory cytokines by Th2 cells, such as IL‐4 and IL‐13, while Th1 cells showed decreased IFN‐γ production, leading to a Th1/Th2 imbalance (Silva‐Pavez et al. 2024). FC revealed that Rh1 significantly curbed the rise in IL‐4^+^CD4^+^ cells in AR mice, while elevating IFN‐γ^+^CD4^+^ cell proportions (Figure 1D,E). Furthermore, Rh1 reduced eosinophil counts (Figure 1F). ELISA results indicated significantly higher levels of serum IgE, eosinophil counts, and Th2 cytokines in NALF of the model group (Figure 1G), which were significantly lowered following Rh1 administration. These findings support the role of Rh1 in mitigating HDM‐induced AR by suppressing nasal mucosal inflammation and correcting the Th1/Th2 imbalance.

### 3.2 Rh1's Role in Reducing Nasal Mucosal Apoptosis and Inflammation in AR Mice

**FIGURE 2:**
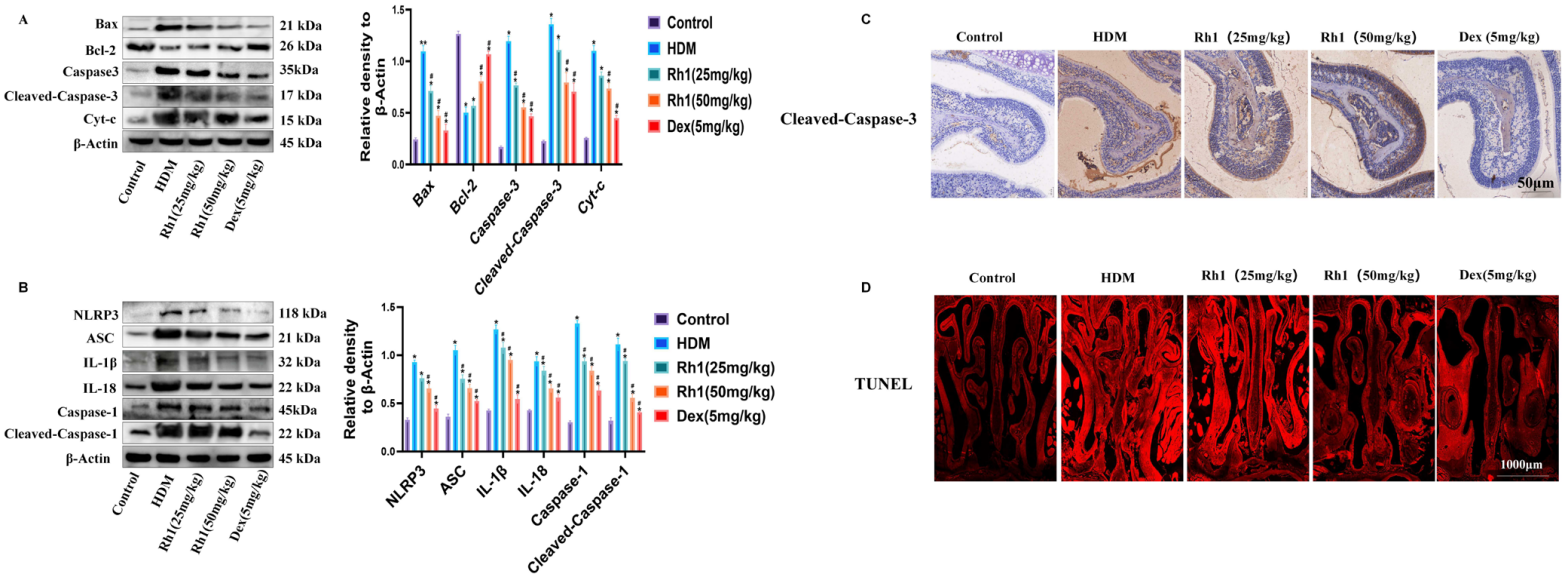
Ginsenoside Rh1 inhibits apoptosis and inflammation in nasal mucosal epithelial cells of AR mice. (A) WB analysis showing the expression of apoptosis‐related proteins in nasal mucosal cells of mice. (B) WB analysis revealing the levels of inflammation‐related proteins in the nasal mucosa of mice. (C) Histological analysis indicating the expression of Cleaved‐Caspase‐3. (D) TUNEL assay results for detection of cell apoptosis in nasal tissue sections. Data are presented as mean ± SD (*n* = 8). *p < 0.05, compared with control group. ^#^ *p* < 0.05 compared with HDM‐challenged group.

To assess Rh1's impact on apoptosis in nasal mucosal epithelial cells, TUNEL staining and WB analyses were employed to detect apoptotic proteins. Rh1 significantly reduced the expression of apoptotic markers, including Cleaved‐Caspase‐3, and Cyt‐c, while increasing the expression of the anti‐apoptotic protein Bcl‐2 (Figure 2A). We also found that, following Rh1 treatment, the expression levels of NLRP3, ASC, IL‐1β, IL‐18, Caspase‐1, and Cleaved‐Caspase‐1 were all significantly reduced (Figure 2B). Immunohistochemical analysis further confirmed a reduction in Cleaved‐Caspase‐3 expression following Rh1 treatment (Figure 2C). The number of TUNEL‐positive apoptotic cells in the Rh1‐treated group's nasal mucosal tissues was significantly lower compared to the model group (Figure 2D). These findings suggest that Rh1 exhibits both anti‐apoptotic and anti‐inflammatory effects in the nasal tissues of AR mice.

### 3.3 Effects of Ginsenoside Rh1 on AR Based on Network Pharmacology

**FIGURE 3:**
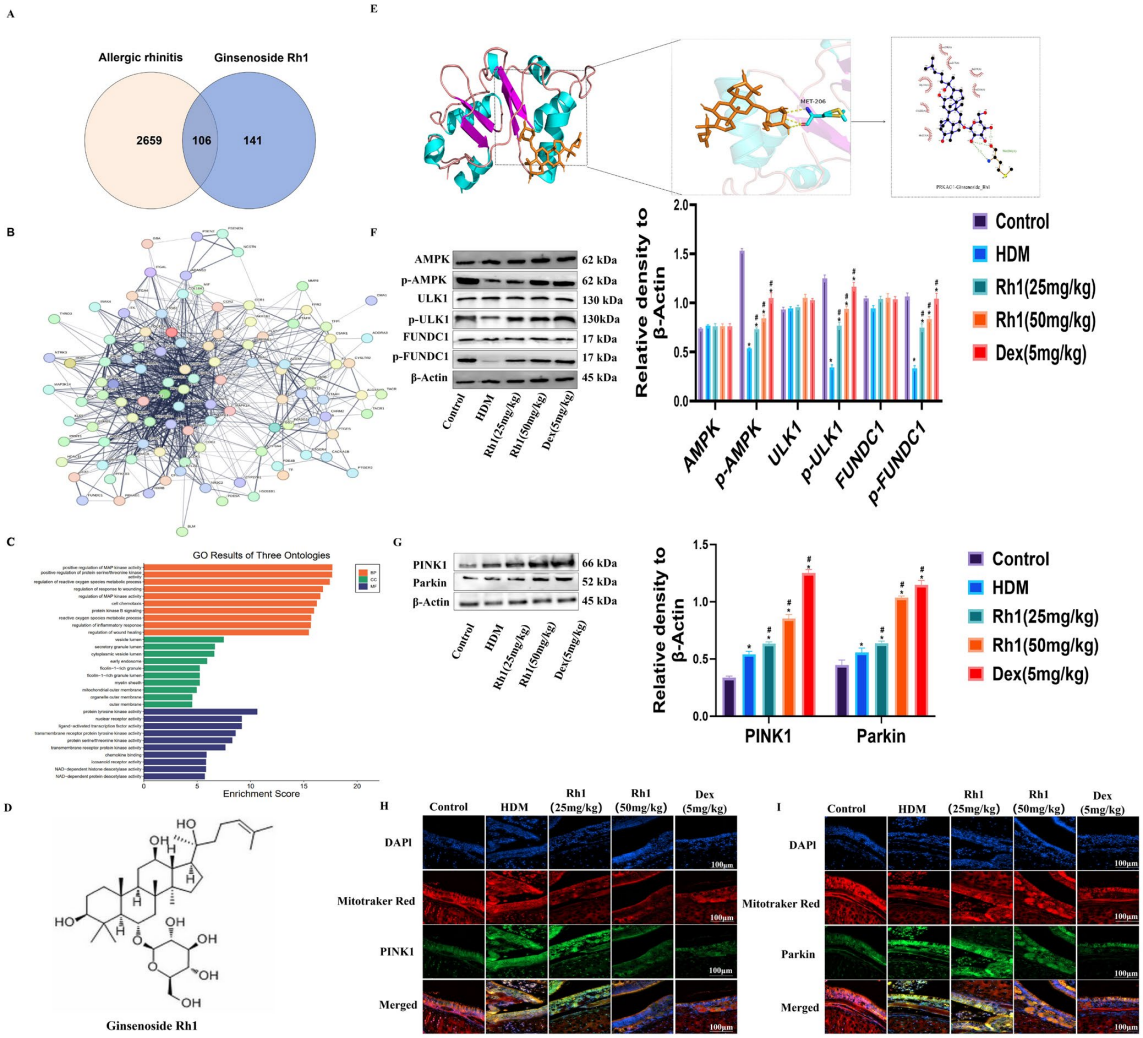
Ginsenoside Rh1 activates the AMPK/ULK1/FUNDC1 pathway to enhance mitochondrial autophagy in AR mice. (A) Venn diagram illustrating the overlap between AR and the targets of Ginsenoside Rh1. (B) PPI network highlighting interactions among key targets, including AMPK (PRKAG1), ULK1, and FUNDC1. (C) GO analysis of the three biological ontologies. (D) The chemical structure of Ginsenoside Rh1. (E) MD of Ginsenoside Rh1 and AMPK. (F) WB analysis demonstrating protein levels of AMPK, ULK1, and FUNDC1, as well as their phosphorylated forms. (G) WB results showing the protein levels of PINK1 and Parkin. (H, I) IF detection of PINK1 and Parkin in the nasal mucosa. Data are presented as mean ± SD (*n* = 8). *p < 0.05, compared with control group. ^#^ *p* < 0.05 compared with HDM‐challenged group.

To explore the molecular mechanisms linking Rh1 to AR, a network pharmacology analysis was performed. Disease targets were identified from GeneCards, OMIM, and DISGENET gene libraries. Monomer structures and SMILES were obtained from PubChem. These targets were then imported into SwissTargetPrediction, PharmMapper, SEA, and SuperPred databases for further analysis, using a probability criterion of greater than zero. After matching and comparison, 106 components and disease‐related cross‐targets were identified using Venny software (Figure 3A). A protein–protein interaction (PPI) network was constructed, revealing the key involvement of AMPK (PRKAG1), ULK1, and FUNDC1 in AR (Figure 3B). Gene Ontology (GO) analysis was also conducted to identify the biological mechanisms and pathways associated with Rh1 (Figure 3C). Docking studies of Rh1 with AMPK (PRKGA1) demonstrated interactions with the amino acid residue MET‐206, with red arc‐shaped markings highlighting crucial interaction sites, such as near amino acid residues Gly218 (A), His215 (A), Gln222 (A), His224 (A), and MET‐206 (A). We examined the chemical structure of Ginsenoside Rh1 (Figure 3D). These insights are pivotal for understanding the molecular interactions that may regulate multiple signaling pathways (Figure 3E), including the AMPK/ULK1/FUNDC1 pathway, potentially significant in AR.

### 3.4 Rh1 Activates Mitochondrial Autophagy in HDM‐Induced AR Mice via the AMPK/ULK1/FUNDC1 Pathway

WB analysis demonstrated that Rh1 treatment upregulated the expression of p‐AMPK, p‐ULK1, and p‐FUNDC1 (Figure 3F), as well as PINK1 and Parkin (Figure 3G) in comparison to the HDM group. IF analysis showed a marked increase in fluorescence intensity of PINK1 and Parkin following Rh1 treatment (Figure 3H,I). Thus, Rh1 may mitigate inflammation by activating mitophagy in HDM‐induced AR mice through the AMPK/ULK1/FUNDC1 pathway.

### 3.5 Rh1 Regulates Mitochondrial Autophagy in HDM‐Treated HNEpCs via the AMPK/ULK1/FUNDC1 Pathway

**FIGURE 4:**
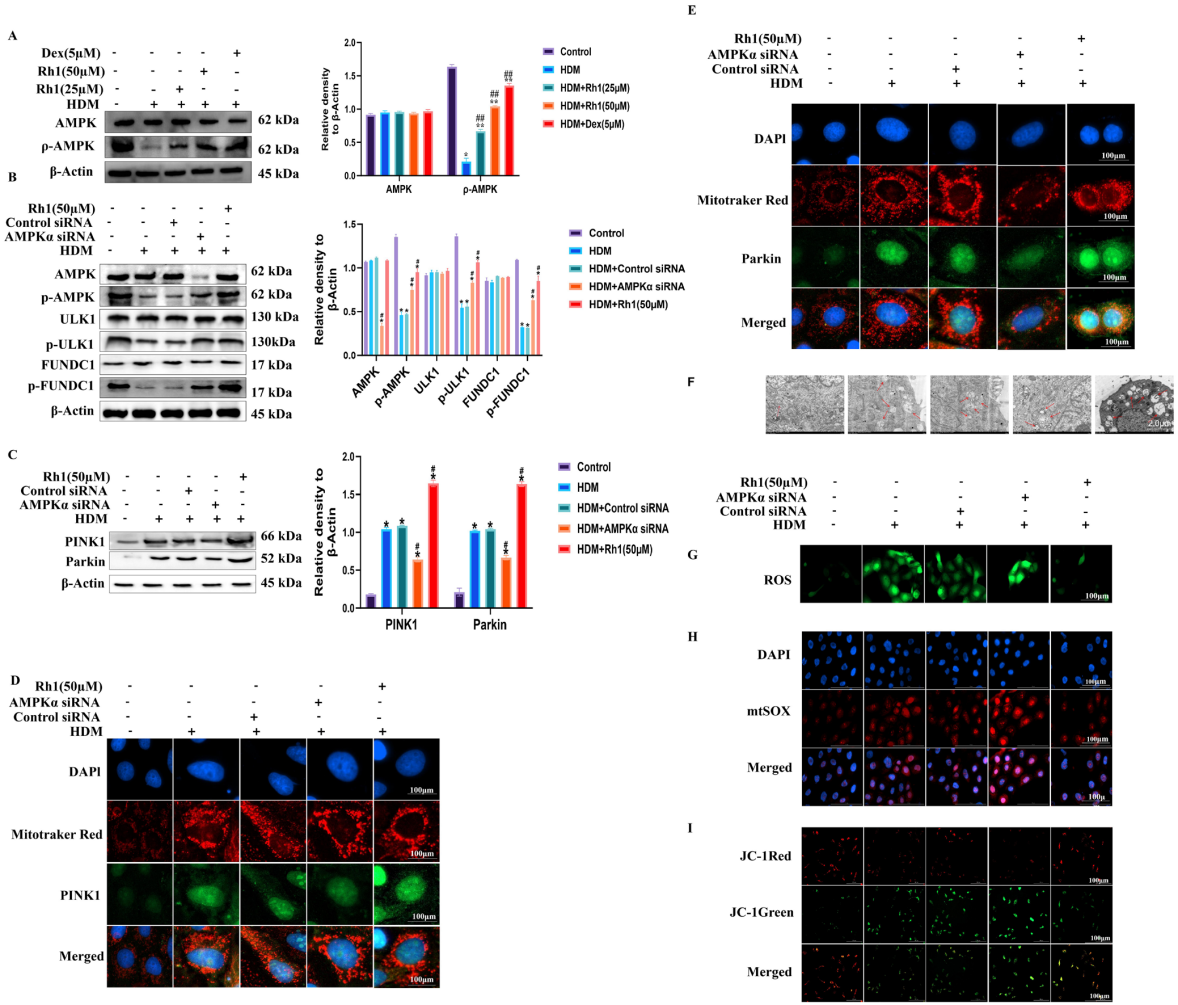
si‐AMPK treatment of HDM‐stimulated HNEpCs cells affects mitophagy. (A) WB analysis assessing the expression of AMPK and its phosphorylated form. (B) WB analysis revealing protein levels of AMPK, ULK1, and FUNDC1, along with their phosphorylated forms. (C) WB analysis of PINK1 and Parkin protein expression. (D, E) Colocalization of MitoTracker Red with PINK1 and Parkin in HNEpCs. (F) TEM analysis of mitochondrial morphology. (G) Detection of ROS using DCFH‐DA. (H) Representative microscopy images and quantification of mitochondrial ROS (MitoSOX) in HNEpCs. (I) JC‐1 dye analysis of mitochondrial membrane potential in HNEpCs post‐HDM treatment. Data are presented as mean ± SD (*n* = 8). *p < 0.05, compared with control group. ^#^ *p* < 0.05compared with HDM‐challenged group.

The regulatory effect of Rh1 on AMPK was explored in vitro. Rh1 treatment significantly increased the phosphorylation of AMPKα in HNEpCs cells (Figure 4A). Subsequent experiments used Rh1 at a concentration of 50 μM. Following AMPK knockdown with siRNA, a reduction in p‐AMPK, p‐ULK1, and p‐FUNDC1 levels was observed in the HDM + AMPKα siRNA group (Figure 4B). WB analysis further confirmed a decrease in PINK1 and Parkin expression after AMPK knockdown (Figure 4C, Figure S1A), and these findings were supported by IF analysis (Figure 4D,E). TEM demonstrated a reduction in the number of autophagosomes following AMPK knockdown with siRNA (Figure 4F). In summary, AMPK knockdown affected the downstream signaling pathway and attenuated the autophagy effect, indicating that Rh1 may influence mitochondrial autophagy in HNEpCs cells through the AMPK/ULK1/FUNDC1 pathway.

### 3.6 Rh1 Inhibits the Production of Mitochondrial Reactive OS and Upregulates Mitochondrial Membrane Potential in HDM‐Treated HNEpCs

HNEpCs were stimulated with HDM to provoke a Th2 immune response, thereby exacerbating OS. This, in turn, can amplify inflammatory reactions, disrupt the nasal mucosal epithelium's protective barrier, and initiate AR. The levels of ROS and Mitochondrial Reactive OS (mtROS) were measured, showing that Rh1 significantly reduced their production (Figure 4G,H). Notably, the inhibitory effect of Rh1 was attenuated when AMPK was knocked down using siRNA. The mitochondrial membrane potential was assessed using the JC‐1 fluorescent probe (Figure 4I). In the control group, uniform red fluorescence was observed. However, post‐HDM stimulation, a decrease in the red‐to‐green fluorescence ratio indicated membrane depolarization. Treatment with Rh1 significantly restored the red/green fluorescence ratio. This effect was also diminished when AMPK was knocked down with siRNA. Thus, Rh1 reduces mtROS generation induced by HDM and enhances mitochondrial membrane potential through the AMPK/ULK1/FUNDC1 pathway, mitigating mitochondrial damage.

### 3.7 Rh1 Regulates NLRP3 Inflammasome and Mitochondrial Apoptosis by Promoting Mitochondrial Autophagy in HDM‐Stimulated HNEpCs

**FIGURE 5:**
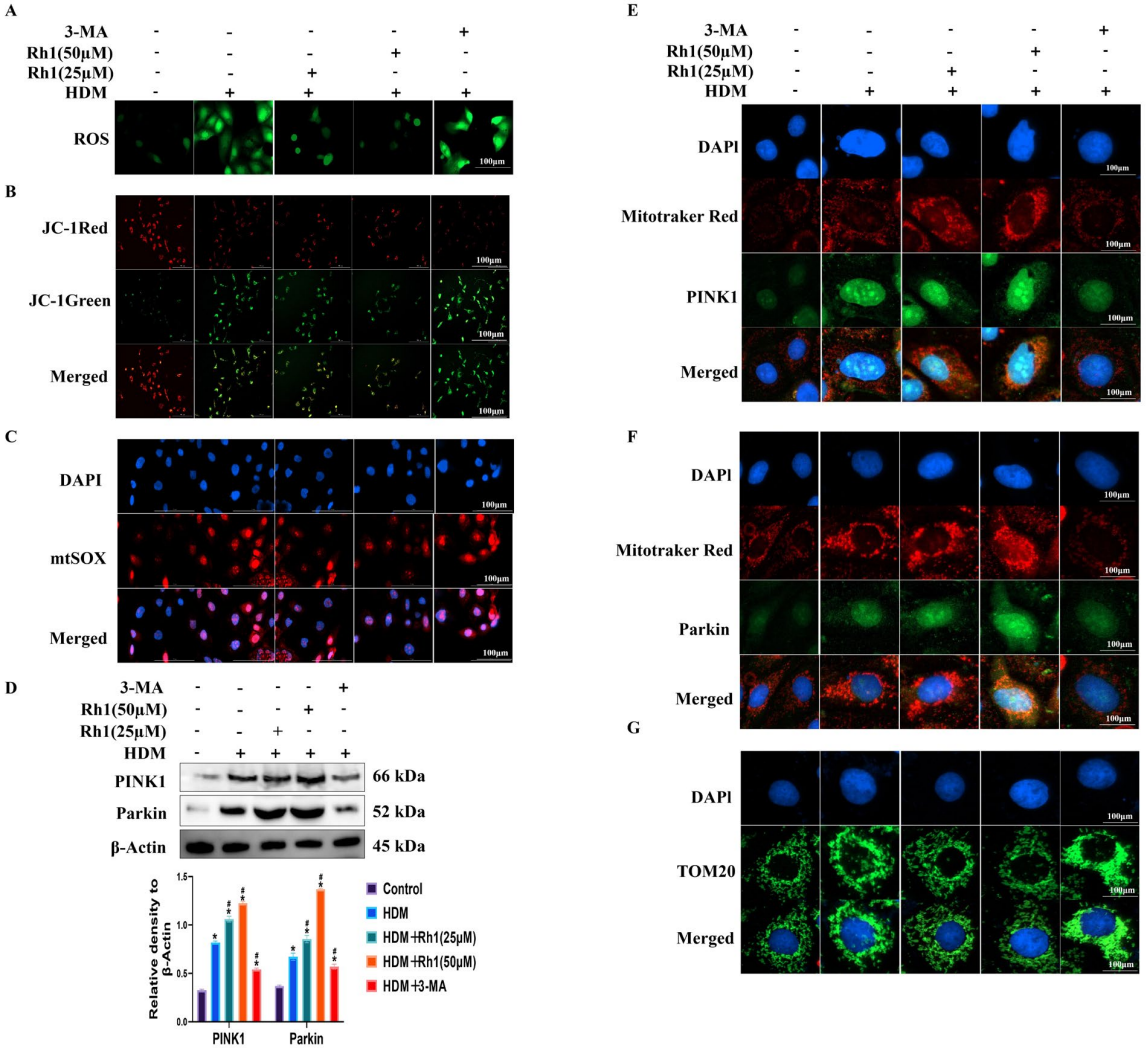
Pretreatment with 3‐MA enhances MitoROS production, NLRP3 inflammasome activation, and apoptosis in HDM‐stimulated HNEpCs by inhibiting mitochondrial autophagy (A) ROS detection using DCFH‐DA. (B) Mitochondrial membrane potential analysis in HNEpCs treated with HDM using JC‐1 dye. (C) Representative micrographs and quantification of mitochondrial ROS (MitoSOX) in HNEpCs. (D) WB analysis of PINK1 and Parkin protein levels. (E, F) Co‐localization of MitoTracker Red with PINK1 and Parkin in HNEpCs. (G) Assessment of mitochondrial morphology in HNEpCs using TOM20. Data are presented as mean ± SD (*n* = 8).*p < 0.05 compared with control group. ^#^ *p* < 0.05 compared with HDM‐challenged group.

**FIGURE 6:**
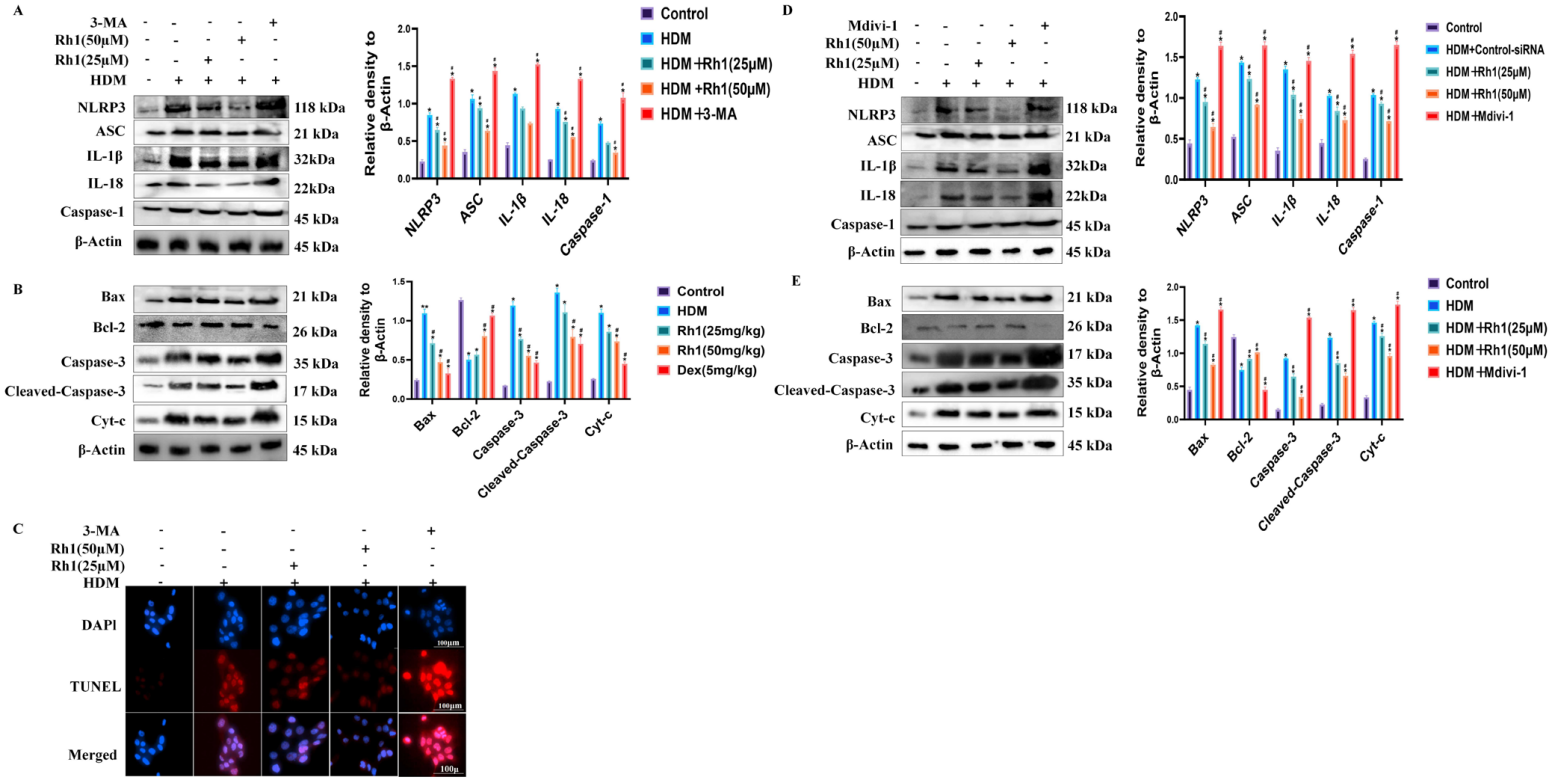
Pretreatment with 3‐MA enhances NLRP3 inflammasome activation, product formation, and apoptosis in HDM‐stimulated HNEpCs. (A, B) WB analysis of apoptosis‐ and inflammation‐related proteins in mouse nasal mucosal cells. (C) Apoptosis detection using the TUNEL method. (D, E) WB analysis of apoptosis and inflammation‐related proteins in nasal mucosal cells. Data are presented as mean ± SD (*n* = 8). *p < 0.05 compared with control group. ^#^ *p* < 0.05 compared with HDM‐challenged group.

3‐MA, a known inhibitor of mitochondrial autophagy, was used to pretreat HNEpCs at 10 μmol/L (Ponda et al. 2023) for 24 h to inhibit mitochondrial autophagy activity. ROS, mtROS, and mitochondrial membrane potential were then assessed. 3‐MA was found to increase ROS and mtROS levels and decrease mitochondrial membrane potential (Figure 5A–C). WB analysis showed that 3‐MA reduced the protein expression of PINK1 and Parkin (Figure 5D, Figure S2A), which was confirmed by IF (Figure 5E,F). Mitochondrial fragmentation was increased under 3‐MA's influence on TOM20 (Figure 5G). These results indicate that Rh1's effects were countered by 3‐MA in HDM‐stimulated HNEpCs. Furthermore, after 3‐MA treatment, the expression of inflammatory proteins, was notably elevated (Figure 6A). In parallel, 3‐MA increased the expression of pro‐apoptotic markers such as Cleaved‐Caspase‐3, and Cyt‐c, while decreasing Bcl‐2 expression (Figure 6B). TUNEL staining corroborated the WB findings regarding apoptosis (Figure 6C). Moreover, using Mdivi‐1, another mitochondrial autophagy inhibitor, yielded similar results to 3‐MA as shown in WB (Figure 6D,E). These findings suggest that Rh1 regulates inflammation and apoptosis through mitochondrial autophagy.

## 4 Discussion

As an allergic disease, allergic rhinitis (AR) severely affects a large proportion of the global population, causing significant inconvenience in patients' daily lives and work (Ponda et al. 2023). AR is an important risk factor for the development of asthma, and the two conditions often coexist. Nasal inflammation in AR patients can impact the lower respiratory tract, leading to airway hyperresponsiveness and inflammation (Brożek et al. 2017).

**FIGURE 7:**
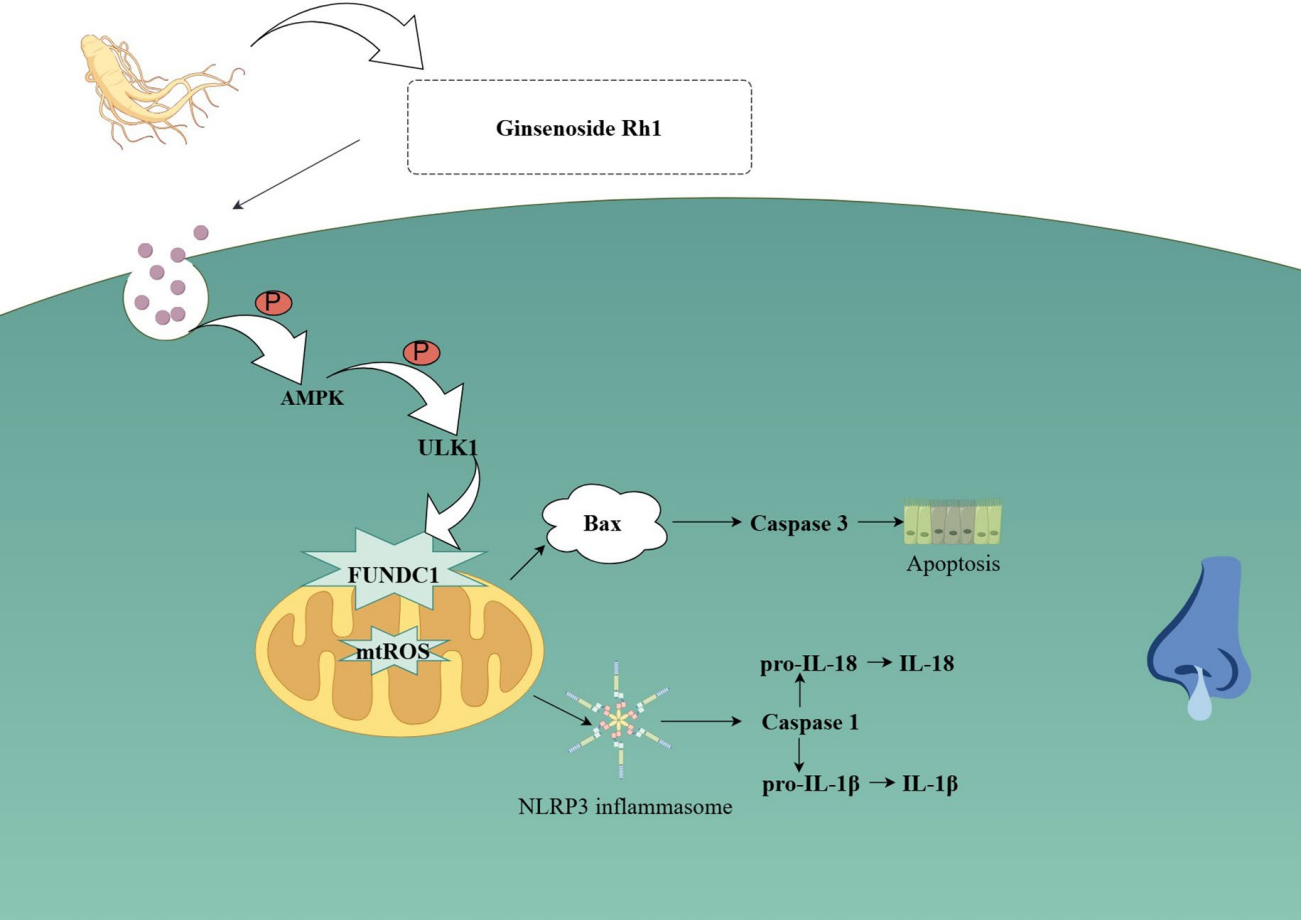
Mechanism diagram. Schematic representation of mitophagy, mitochondrial ROS, NLRP3 inflammasome activation, and apoptosis mechanisms in AR. HDM stimulation induces mitochondrial damage in HNEpCs, resulting in mitochondrial ROS production and NLRP3 inflammasome activation. Ginsenoside Rh1 promotes autophagy via the AMPK/ULK1/FUNDC1 pathway, facilitating mitochondrial repair, reducing mitochondrial ROS, and attenuating NLRP3 inflammasome activation, ultimately preventing AR. Rh1 enhances AMPK activation, modulates AMPK phosphorylation, inhibits mitochondrial fission, and consequently suppresses the TXNIP/NLRP3 pathway.

Ginsenoside Rh1 is a triterpenoid saponin and one of the main active components in ginseng (Piao et al. 2020). Accumulating evidence indicates that ginsenosides exhibit significant anti‐inflammatory and antioxidant activities in various diseases (Niu et al. 2024). Rh1 has been shown to inhibit apoptosis in nasal mucosal epithelial tissue and suppress the expression of apoptosis‐related and inflammasome‐related proteins, such as NLRP3, ASC, IL‐1β, IL‐18, Caspase‐1, and Cleaved‐Caspase‐1. Su et al. (2021) reported that in type 2 diabetic nephropathy, Ginsenoside Rh1 can inhibit inflammation and apoptosis through the AMPK/PI3K/AKT pathway, thereby improving the disease. However, the regulatory mechanisms of Rh1 on the AMPK/PI3K/AKT pathway in AR remain unclear (Figure 7).

Related studies have shown that AMPK, often referred to as the “cellular energy regulator,” plays a central role in regulating cellular energy metabolism. When mitochondrial damage is severe, AMPK can sense mitochondrial damage signals and phosphorylate specific amino acid residues of ULK1, thereby activating ULK1. ULK1 then translocates to the mitochondria, regulating mitophagy and laying the foundation for its occurrence. In a model of doxorubicin‐induced cardiomyopathy and fibrosis, activating the AMPK/ULK1/FUNDC1 pathway reduces ROS levels, improves mitochondrial membrane potential, and increases mitophagy, thereby ameliorating cardiomyopathy (Zhao et al. 2024). The role of AMPK in regulating ULK1/FUNDC1‐mediated mitophagy to mitigate disease has also been confirmed in other studies. Research by Yang et al. (2023) suggests that inhibiting mitophagy by inactivating the AMPK/ULK1/FUNDC1 pathway leads to energy metabolism disorders and causes hippocampal neuronal damage in mice. Studies by Cai et al. (2022) have shown that activating FUNDC1 through the AMPKα1/ULK1 pathway reduces ROS production and increases mitophagy, protecting the endothelial barrier function and integrity of cardiac microvascular endothelial cells (CMECs) and thereby maintaining cardiac microvascular structure and function. In this study, using network pharmacology and molecular docking techniques, we identified 106 cross‐targets and found that Rh1 interacts with specific amino acid residues of AMPK (PRKGA1), such as MET‐206. Our study demonstrates that treatment with Ginsenoside Rh1 increases the phosphorylation levels of AMPK, ULK1, and FUNDC1 compared to the model group, indicating that Rh1 activates the AMPK/ULK1/FUNDC1 signaling pathway.

Related studies have found that AMPK is associated with the activation of the NLRP3 inflammasome. The phosphorylation of AMPK promotes the activation of the NLRP3 inflammasome (Bullón et al. 2016). Studies have shown that Ginsenoside Rg1 can inhibit the NLRP3 inflammasome by inducing autophagy, thereby alleviating acute liver injury. The NLRP3 inflammasome is a crucial multiprotein complex within the cell, composed of the NLRP3 receptor protein, the ASC adaptor protein, and the Caspase‐1 protease. Upon activation of this complex, the precursor of Caspase‐1 undergoes proteolytic cleavage, forming an active enzyme that catalyzes the conversion of pro‐IL‐1β to mature cytokines and their release outside the cell. Huang et al. (2021) have shown that enhancing autophagy through the AMPK/mTOR/ULK1 signaling pathway can alleviate NLRP3 inflammasome‐related neuroinflammation. Other studies have shown that inhibiting the activation of the NLRP3 inflammasome can reduce allergic and inflammatory responses in AR mouse models (Bai et al. 2022). Similarly, we observed that Rh1 prevented the activation of the NLRP3 inflammasome in HDM‐induced HNEpCs cells, and the use of 3‐MA also inhibited the activation of the NLRP3 inflammasome, with similar effects seen with Mdivi‐1. These findings reveal the regulatory molecular mechanisms of Rh1 in AR. Excessive ROS can activate the NLRP3/IL‐1β pathway, and inhibiting ROS production can alleviate acute lung injury (Liu et al. 2024).

Mitophagy serves as a crucial pathway for suppressing inflammation. The normal function of mitochondria is vital for cellular energy metabolism and homeostasis. When mitochondria are damaged, it leads to increased production of reactive oxygen species (ROS) and calcium ion homeostasis imbalance within the cell (Jiang et al. 2022). These changes can indirectly activate inflammatory signaling pathways. Mitophagy helps maintain mitochondrial function and cellular homeostasis by clearing damaged mitochondria, thereby reducing inflammatory responses (Liu et al. 2022). When mitochondria are stressed (e.g., under oxidative stress, hypoxia, or loss of mitochondrial membrane potential), they produce damage signals. PINK1 accumulates on the outer membrane of damaged mitochondria and recruits Parkin through phosphorylation (Nguyen et al. 2016). Parkin further phosphorylates mitochondrial outer membrane proteins, marking the damaged mitochondria for clearance. By removing damaged mitochondria, mitophagy helps maintain cellular energy metabolism balance (Narendra and Youle 2024). Studies by Gong et al. (2025) have shown that Ginsenoside Rh1 accelerates mitophagy, promotes mitochondrial fusion, inhibits mitochondrial fission, and reduces myocardial ischemic injury and oxidative stress in hypoxic‐damaged cardiomyocytes. We found that, in vitro, Rh1 effectively increased mitochondrial membrane potential and mitochondrial autophagy proteins in HDM‐induced HNEpCs cells, while reducing apoptotic proteins such as Bax, Caspase‐3, Cleaved‐Caspase‐3, and Cyt‐c, and increasing Bcl‐2 expression. Similarly, an increase in Tom20 was observed. Rh1 inhibited the production of ROS and mtROS in nasal mucosal tissue and HNEpCs cells, reducing the expression of inflammatory proteins.

In this study, Rh1 was able to activate AMPK. The phosphorylation regulation of ULK1 and FUNDC1 was attenuated by siRNA knockdown of AMPKα, which inhibited the loss of mitochondrial membrane potential and the increase in ROS production in HNEpCs cells. This, in turn, prevented the activation of PINK1 mediated by oxidative stress and the recruitment of Parkin to damaged mitochondria. Knockdown of AMPK also weakened the increase in PINK1 and Parkin in HNEpCs cells induced by HDM, and TEM revealed a reduction in autophagy. 3‐MA, an autophagy inhibitor, had a similar effect to AMPKα knockdown. Based on the above data, it was confirmed that AMPK knockdown inhibited the Rh1‐activated AMPK/ULK1/FUNDC1 pathway, which affected mitophagy and subsequently led to an increase in inflammation.

The above research results further support our conclusion that Ginsenoside Rh1 inhibits the NLRP3 inflammasome and mitochondrial ROS production through AMPK/ULK1/FUNDC1‐mediated mitophagy, thereby ameliorating AR‐induced nasal mucosal inflammation in AR mice. These findings provide a novel molecular regulatory mechanism and a potential therapeutic target for the improvement of AR.

## Author Contributions

J.W., Y.Z., and J.C. contributed to the study design, data collection and analysis, and manuscript writing. J.Y., L.D., Y.Y., Y.Z., and Y.J. contributed to study design and paper revision. C.W. and G.Y. was responsible for the conception, funding, and final version. All the authors read and approved the final manuscript.

## Ethics Statement

All of the animal experiments in this work have received animal ethical permission from the Yanbian University of Medicine Experimental Animal Center, YanJi, China. All experimental protocols were performed in accordance with the ARRIVE guidelines (YD20230620006).

## Consent

Written informed consent for publication was obtained from all participants.

## Conflicts of Interest

The authors declare no conflicts of interest.

## Supporting information


Data S1.

## Data Availability

All data generated or analyzed during this study are included in this published article.
