# Supplementary material for: Ginsenoside Rh1 Alleviates Allergic Rhinitis by Mediating Mitochondrial Autophagy via Activation of the AMPK/ULK1/FUNDC1 Pathway
Source: Food Sci Nutr. 2025 Jun 17;13(6):e70464. doi: 10.1002/fsn3.70464 (PMC12173953; doi:10.1002/fsn3.70464)
Supplement: Supplementary file 1 — Data S1. [file FSN3-13-e70464-s001.docx]

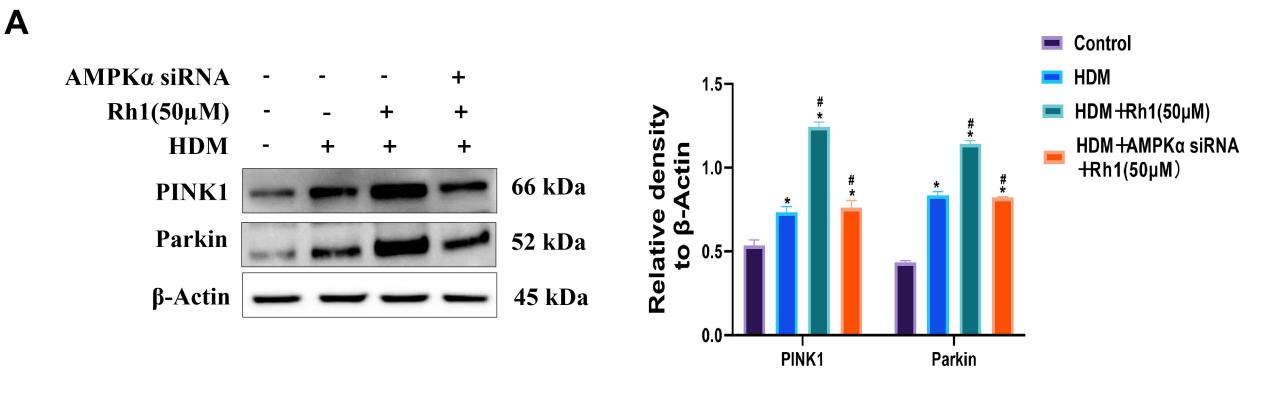


**FIGURE S1 si-AMPK Treatment of HDM-Stimulated HNEpCs Cells Affects Mitophagy.（A）** The levels of mitophagy proteins PINK1 and Parkin in HNEpCs induced by HDM were detected by Western Blotting. Data are presented as mean±SD (n = 8). **p*<0.05 compared with control group. **^#^***p*<0.05 compared with HDM-challenged group.


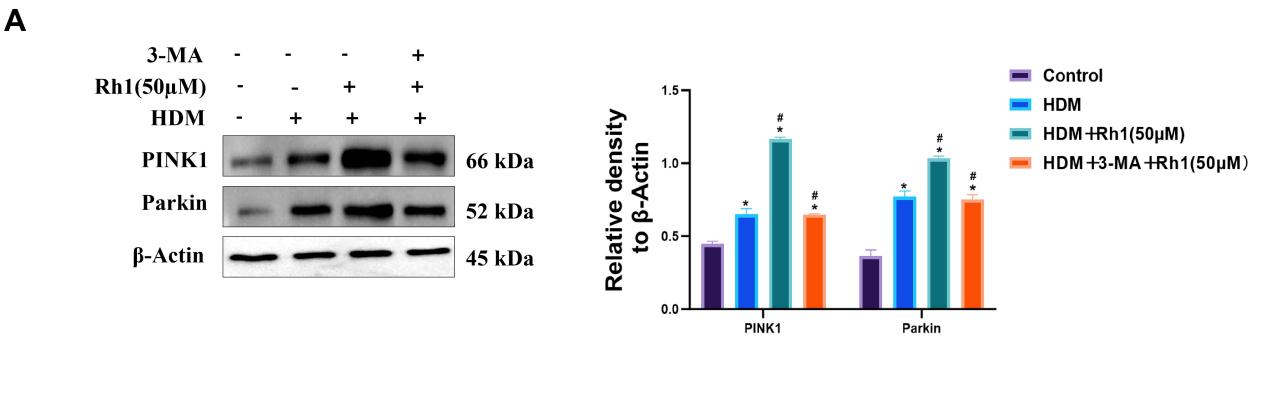


**FIGURE S2 3-MA Treatment of HDM-Stimulated HNEpCs Cells Affects Mitophagy. （A）**The levels of mitophagy proteins PINK1 and Parkin in HNEpCs induced by HDM were detected by Western Blotting. Data are presented as mean±SD (n = 8). **p*<0.05 compared with control group. **^#^***p*<0.05 compared with HDM-challenged group.
